# Prognostic implications of resting distal coronary-to-aortic pressure ratio compared with fractional flow reserve: a 10-year follow-up study after deferral of revascularisation

**DOI:** 10.1007/s12471-020-01365-6

**Published:** 2020-01-21

**Authors:** G. W. M. Wijntjens, T. P. van de Hoef, M. Meuwissen, M. Echavarría-Pinto, T. Murai, V. E. Stegehuis, K. T. Koch, S. A. Chamuleau, M. Voskuil, R. J. de Winter, J. G. P. Tijssen, J. J. Piek

**Affiliations:** 1 Heart Centre, Amsterdam Universitair Medische Centra, locatie Academic Medical Centre, Amsterdam, The Netherlands; 2 Department of Cardiology, Amphia Hospital, Breda, The Netherlands; 3 Hospital General ISSSTE, Facultad de Medicina, Universidad Autónoma de Querétaro, Querétaro, Mexico; 4 Department of Cardiology, University Medical Centre Utrecht, Utrecht, The Netherlands

**Keywords:** Coronary artery disease, Fractional flow reserve, Resting distal-to-aortic pressure ratio, Major adverse cardiac events

## Abstract

**Introduction:**

The distal coronary-to-aortic pressure ratio (*P*_d_/*P*_a_) is a non-hyperaemic physiological index to assess the functional severity of coronary stenoses. Studies comparing *P*_d_/*P*_a_ with fractional flow reserve (FFR) show superior diagnostic efficiency for myocardial ischaemia. Nevertheless, a direct comparison regarding long-term clinical outcomes is still not available. The present observational study compared the prognostic value of *P*_d_/*P*_a_ and FFR for major adverse cardiac events (MACE) during a 10-year follow-up period after deferral of revascularisation.

**Methods:**

Between April 1997 and September 2006, we evaluated 154 coronary stenoses (154 patients) in which revascularisation was deferred with intracoronary pressure and flow measurements during the resting and hyperaemic state. Long-term follow-up (median: 11.8 years) was performed to document the occurrence of MACE, defined as a composite of cardiac death, myocardial infarction and target vessel revascularisation.

**Results:**

The study population comprised angiographically intermediate coronary stenoses, with a mean diameter stenosis of 53 ± 8%, and intermediate physiological severity with a median FFR of 0.82 (Q1, Q3: 0.76, 0.88). The association of *P*_d_/*P*_a_ with long-term MACE was similar to that of FFR [FFR-standardised hazard ratio (sHR): 0.77, 95% confidence interval (CI): 0.61–0.98; *P*_d_/*P*_a_-sHR: 0.80, 95% CI: 0.67–0.96]. In the presence of disagreement between *P*_d_/*P*_a_ and FFR, normal *P*_d_/*P*_a_ was generally associated with high coronary flow reserve (CFR) and a favourable clinical outcome, whereas abnormal *P*_d_/*P*_a_ was generally associated with CFR around the ischaemic cut-point and an impaired clinical outcome, regardless of the accompanying FFR value.

**Conclusion:**

The present study suggests that *P*_d_/*P*_a_ provides at least equivalent prognostic value compared with FFR. When *P*_d_/*P*_a_ disagreed with FFR, the baseline index conferred superior prognostic value in this study population.

**Electronic supplementary material:**

The online version of this article (10.1007/s12471-020-01365-6) contains supplementary material, which is available to authorized users.

## What’s new?

- The distal coronary-to-aortic pressure ratio (*P*_d_/*P*_a_) demonstrates a continuous and independent relationship with subsequent long-term clinical outcomes.
- The prognostic value of *P*_d_/*P*_a_ is at least equivalent to that of fractional flow reserve (FFR), but exceeds FFR as a risk stratification tool at their contemporary clinical cut-off values.
- When *P*_d_/*P*_a_ disagreed with FFR, *P*_d_/*P*_a_ conferred superior prognostic value in this study population.

## Introduction

Guidelines on coronary revascularisation recommend the hyperaemia-dependent fractional flow reserve (FFR) for clinical decision-making, yet its clinical adoption has remained limited. As a corollary, the resting distal coronary-to-aortic pressure ratio (*P*_d_/*P*_a_) and the instantaneous wave-free ratio (iFR) were (re-)introduced with the aim of simplifying the use of coronary physiology in daily clinical practice by enabling its assessment during resting conditions [1–3]. Although baseline indices and FFR show equal diagnostic accuracy for positron emission tomography-derived flow abnormalities [4, 5], studies using invasive Doppler-derived coronary flow reserve (CFR) as the reference standard documented superior identification of flow abnormalities by *P*_d_/*P*_a_ and iFR over FFR [3, 6, 7]. Doppler-derived CFR is considered a critical determinant of myocardial ischaemia and has relevant prognostic value in the setting of stable ischaemic heart disease [8, 9]. Accordingly, two landmark randomised clinical trials demonstrated that an iFR-guided revascularisation strategy is non-inferior to a FFR-guided revascularisation strategy in terms of clinical outcome at 1‑year follow-up [10, 11]. However, the long-term prognostic value of *P*_d_/*P*_a_ has yet not been compared to that of FFR. Therefore, the aim of the present study was to compare the prognostic value of *P*_d_/*P*_a_, with that of FFR for major adverse cardiac events (MACE) during a 10-year follow-up period after deferral of revascularisation.

## Methods

### Data source

Between April 1997 and September 2006, we prospectively enrolled patients with angina referred for evaluation of ≥1 intermediate coronary stenosis (40%–70% diameter stenosis at visual assessment) in a series of study protocols [12–14] that included intracoronary pressure and flow measurements during resting and hyperaemic conditions. We excluded patients with ostial stenoses, serial stenoses, severe renal function impairment, significant left main stenosis, atrial fibrillation, myocardial infarction <6 weeks before screening, prior coronary artery bypass surgery (CABG), or visible collateral development to the perfusion territory of interest. The study protocols complied with the Declaration of Helsinki and were approved by the institutional ethics committee. All patients gave written informed consent.

### Study procedures and subsequent treatment

Coronary angiography was performed according to standard practice. Quantitative coronary angiography analyses were performed offline using validated software (QCA-CMS version 3.32, MEDIS, Leiden, The Netherlands). Intracoronary nitroglycerin (0.1 µg) was given prior to invasive measurements. Intracoronary pressure and flow was measured with 0.014″ pressure and Doppler sensor-equipped guide wires (Philips-Volcano, San Diego, CA, USA) during resting and hyperaemic conditions. Hyperaemia was induced by an intracoronary bolus of adenosine (20–40 µg). Revascularisation was performed at the operator’s discretion, and decisions on further treatment and medication during follow-up were entirely left to the discretion of the treating cardiologist [8, 15].

### Long-term follow-up

Three-, 6‑, 12-month and long-term follow-up was performed by clinical visit or telephone contact to document the occurrence of MACE, defined as the composite of cardiac death, acute myocardial infarction attributable to the target vessel, and clinically driven revascularisation of the target-vessel by CABG or percutaneous coronary intervention (PCI). All patient-reported events were verified and adjudicated after evaluating hospital records or contacting the treating physicians.

### Haemodynamic data analysis

The physiological indices were defined as:

- FFR = mean *P*_d_/mean *P*_a_ (hyperaemia)
- *P*_d_/*P*_a_ = mean *P*_d_/mean *P*_a_ (resting conditions)
- CFR = hyperaemic average peak flow velocity (APV)/baseline APV

**Fig. 1:**
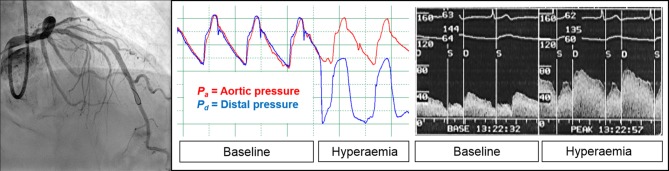
Example of intracoronary pressure and flow measurements. Resting distal-to-aortic pressure ratio (*P*_d_/*P*_a_) is defined as the distal coronary pressure to aortic pressure ratio during baseline conditions. Fractional flow reserve is defined as the distal coronary pressure to aortic pressure ratio during hyperaemic conditions induced by hyperaemic agents. Coronary flow reserve is defined as the coronary flow velocity during hyperaemia to coronary flow velocity during baseline ratio measured distal from the stenosis of interest

For dichotomous evaluations, FFR ≤ 0.80 [16], and *P*_d_/*P*_a_ ≤ 0.92 [2] were considered abnormal (Fig. 1).

### Statistical analysis

In the presence of multiple stenoses of intermediate severity, one was randomly marked the index stenosis and used for subsequent analyses. Normality of the distribution and homogeneity of variances were tested with the Shapiro-Wilk statistic and Levene’s test, respectively. Continuous variables are presented as mean ± standard deviation or median [1st, 3rd quartile (Q1, Q3)], and compared with Student *t*-test or Mann-Whitney U‑test. Categorical variables are presented as frequency (percentage) and compared with Fisher exact test. The prognostic value of FFR and *P*_d_/*P*_a_ for 10-year MACE was assessed using Cox regression analyses, adjusted for the effect of relevant clinical characteristics. The best-fit model for adjustment was identified using Akaike’s information criterion, where candidate covariates were: clinical characteristics (Tab. 1) and the interrogated vessel. All Cox proportional-hazards models were preceded by verification of the proportional hazard assumption using Schoenfeld’s residuals. Results are presented as standardised hazard ratios (sHRs) and their 95% confidence intervals (CIs), estimated from the Cox proportional-hazard models by exponentiating the β‑coefficient multiplied by the SD[exp(β × SD)]. Next, according to their clinical cut-off values, 10-year MACE rates for normal and abnormal FFR and *P*_d_/*P*_a_ were estimated using the Kaplan-Meier (KM) method, and differences were tested with the Wilcoxon-Breslow-Gehan test of equality (Breslow *p*). Finally, KM estimates of 10-year MACE were evaluated across the four quadrants of *P*_d_/*P*_a_ and FFR (dis)agreement, where differences were evaluated with Breslow *p*. A *p*-value below the 2‑sided α‑level of 0.05 was considered statistically significant. The STATA 13.1 statistical software package (StataCorp, College Station, TX, USA) was used for all calculations.

**Table 1:** Demographic, angiographic and physiological characteristics

Number				*n* = 154
*Demographics*				
	Age, years			61 ± 11
	Male			110 (71)
*Risk factors for coronary artery disease*				
	Hypertension			60 (39)
	Hyperlipidaemia			89 (58)
	Positive family history			76 (49)
	Cigarette smoking			48 (31)
	Diabetes mellitus			24 (16)
	Prior myocardial infarction			57 (37)
	Prior PCI			34 (22)
*Medication at hospital admission*				
	Beta-blocker			120 (78)
	Nitrates			110 (71)
	Calcium antagonists			101 (66)
	ACE inhibitors			28 (18)
	Statins			87 (56)
	Acetylsalicylic acid			149 (97)
*Angiographic characteristics*				
	Reference vessel diameter, mm			2.9 ± 0.6
	Diameter stenosis, %			53 ± 8
	Minimal lumen diameter			1.4 ± 0.4
*Physiological characteristics*				
	FFR			0.82 (0.76, 0.88)
	FFR when FFR ≤ 0.80 (*n* = 67)			0.74 (0.70, 0.78)
	FFR when FFR > 0.80 (*n* = 87)			0.88 (0.84, 0.92)
	*P*_d_/*P*_a_			0.94 (0.91, 0.97)
	*P*_d_/*P*_a_ when *P*_d_/*P*_a_ ≤ 0.92 (*n* = 53)			0.90 (0.86, 0.91)
	*P*_d_/*P*a when *P*_d_/*P*_a_ > 0.92 (*n* = 101)			0.97 (0.94, 0.98)
	CFR			2.5 (2.1, 2.9)
	CFR when CFR < 2.0 (*n* = 31)			1.8 (1.5, 1.9)
	CFR when CFR ≥ 2.0 (*n* = 123)			2.6 (2.3, 3.1)

Values are number (%), mean ± standard deviation, median (1st, 3rd quartile)

*FFR* fractional flow reserve; *P*_*d*_*/P*_*a*_ resting distal coronary-to-aortic pressure ratio; *CFR* coronary flow reserve; *PCI* percutaneous coronary intervention; *ACE* angiotensin-converting-enzyme

## Results

### Patient population

A total of 228 patients (299 stenoses) were included. Revascularisation was deferred in 159 patients, in 154 of whom (183 stenoses) resting and hyperaemic coronary pressure and flow velocity data and long-term follow-up were available. In patients with multiple stenoses, one was chosen at random for MACE analyses, which consequently included 154-patients with 154 stenoses. Median follow-up in these patients was 11.8 years (Q1, Q3: 10.0, 13.3 years). Mean age was 60 ± 11 years and 71% of patients were male. Additional baseline characteristics are depicted in Tab. 1.

### Angiographic and physiological measurements

**Fig. 2:**
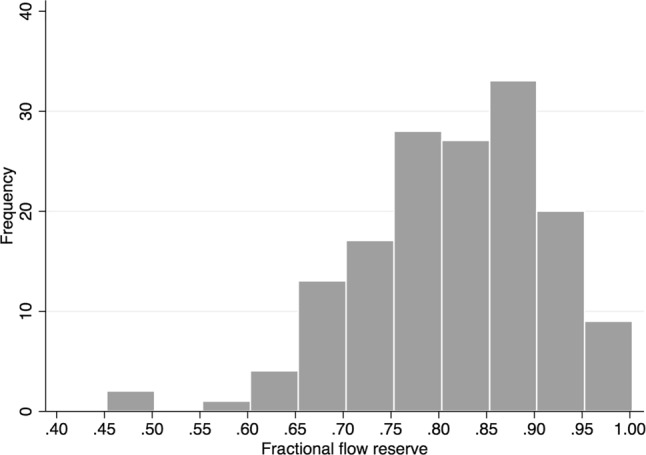
Distribution of fractional flow reserve values across the study population

The study population comprised angiographically intermediate stenoses, with a mean diameter stenosis of 53 ± 8%, and intermediate physiological severity with a median FFR of 0.82 (Q1, Q3: 0.76, 0.88) (Fig. 2), median *P*_d_/*P*_a_ of 0.94 (Q1, Q3: 0.91, 0.97) and median CFR of 2.5 (Q1, Q3: 2.1, 2.9). Stenoses were haemodynamically significant in 43.5% and 34.4% of cases according to FFR and *P*_d_/*P*_a_, respectively. Angiographic and physiological characteristics are depicted in Tab. 1.

### Clinical outcome after deferral of revascularisation stratified by FFR and P_d_/P_a_

**Fig. 3:**
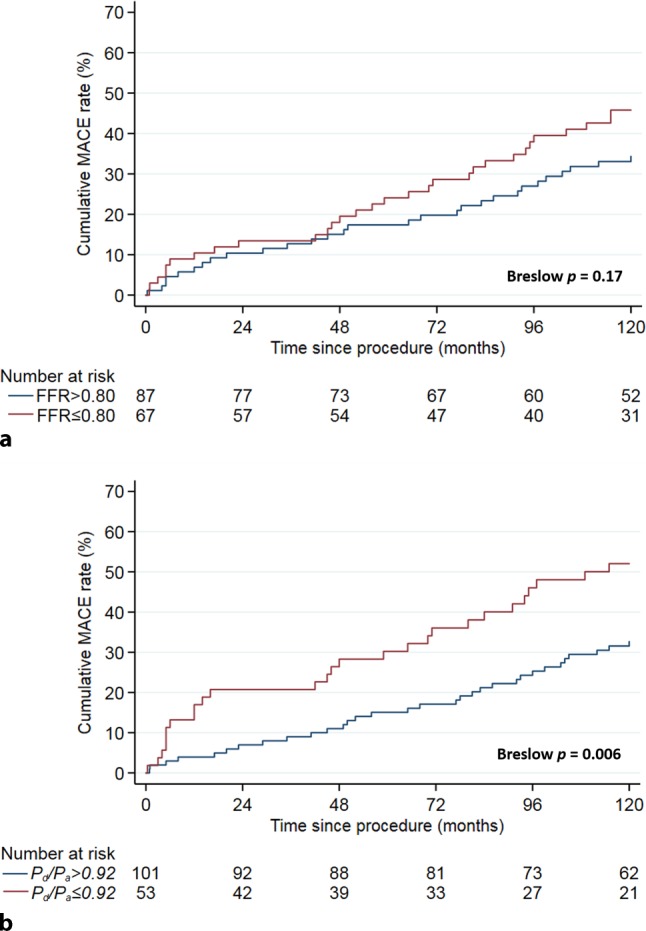
Kaplan-Meier estimates of major adverse cardiac event (*MACE*) rate during 10 years of follow-up, stratified by: **a** fractional flow reserve (*FFR*); FFR ≤ 0.80 was considered abnormal. **b** Resting distal coronary-to-aortic pressure ratio (*P*_d_/*P*_a_). *P*_d_/*P*_a_ ≤ 0.92 was considered abnormal

The best-fit model for adjustment included angiotensin-converting-enzyme inhibitor use, the presence of diabetes mellitus and age. Cox regression analysis adjusted for these variables demonstrated that FFR and *P*_d_/*P*_a_ were both significantly and equivalently associated with long-term MACE [FFR-sHR: 0.77 (95% CI: 0.61–0.98), *p* = 0.033; *P*_d_/*P*_a_-sHR: 0.80 (95% CI: 0.67–0.96), *p* = 0.014] (Tab. 2). Fig. 3 shows the KM curves for cumulative MACE up to 10 years of follow-up according to normal or abnormal results for FFR and *P*_d_/*P*_a_. At 10-year follow-up, the KM estimate of MACE was not significantly different for stenoses with abnormal versus normal FFR (FFR ≤ 0.80: 45.8% vs FFR > 0.80: 34.4%; Breslow *p* = 0.17: Fig. 3a). In contrast, the KM estimate of MACE at 10-year follow-up for stenoses with abnormal *P*_d_/*P*_a_ was significantly higher than for stenoses with normal *P*_d_/*P*_a_ (*P*_d_/*P*_a_ ≤ 0.92: 52.0% vs *P*_d_/*P*_a_ > 0.92 32.7%; Breslow *p* = 0.006: Fig. 3b).

**Table 2:** Univariate and adjusted Cox regression analyses for long-term major adverse cardiac events

Overall study population (*n* = 154)					
Univariate analysis			Adjusted analysis^a^		
Variable	sHR (95%CI)	*p*-value	Variable	sHR (95%CI)	*p*-value
*P*_d_/*P*_a_	0.76 (0.63–0.91)	0.003	*P*_d_/*P*_a_	0.80 (0.67–0.96)	0.014
FFR	0.76 (0.59–0.98)	0.034	FFR	0.77 (0.61–0.98)	0.033

Data presented as standardised hazard ratio and its 95% confidence interval

^a^Adjusted for angiotensin-converting enzyme inhibitor, the presence of diabetes mellitus and age

*sHR* standardised hazard ratio; *P*_d_/*P*_a_ resting distal coronary-to-aortic pressure ratio; *FFR* fractional flow reserve

### Clinical outcome according to discordance between FFR and P_d_/P_a_

**Fig. 4:**
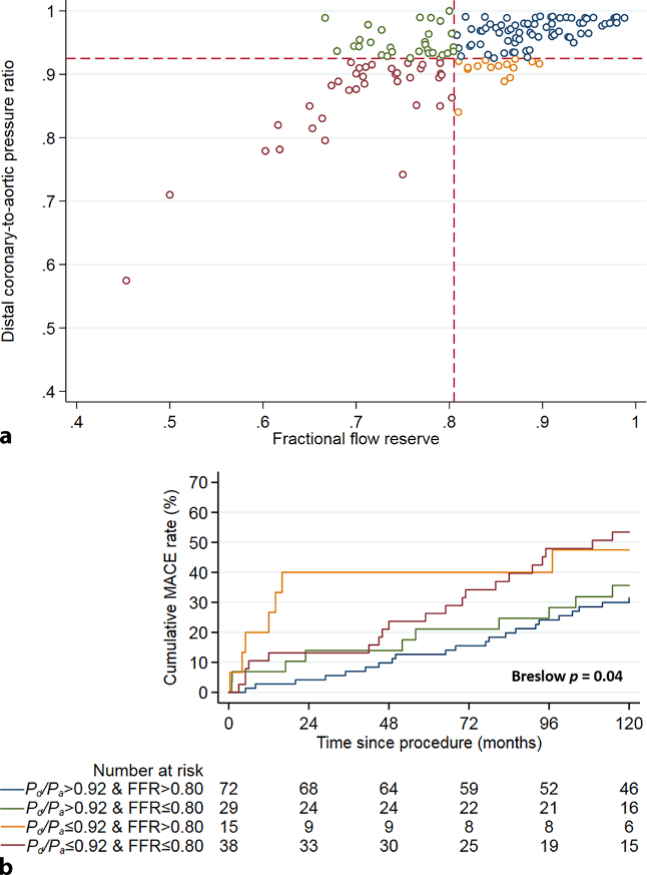
**a**, **b** Discordance between resting distal coronary to aortic pressure ratio (*P*_d_/*P*_a_) and fractional flow reserve (*FFR*). **a** Scatterplot of *P*_d_/*P*_a_ and FFR. The *dotted lines* represent the respective cut-off values for *P*_d_/*P*_a_ (0.92), and FFR (0.80). **b** Kaplan-Meier estimates of major adverse cardiac event (*MACE*) rate during 10 years of follow-up, stratified by *P*_d_/*P*_a_-FFR discordance

*P*_d_/*P*_a_ agreed with FFR in 71% of stenoses (110 out of 154), of which 25% of the stenoses (38 out of 154) were concordant abnormal, and 47% of stenoses (72 out of 154) were concordant normal. *P*_d_/*P*_a_ disagreed with FFR in 29% of stenoses (44 out of 154), and was characterised by abnormal FFR and normal *P*_d_/*P*_a_ in 19% of stenoses (29 out of 154) and by normal FFR and abnormal *P*_d_/*P*_a_ in 10% of stenoses (15 out of 154) (Fig. 4a). Fig. 4b depicts the KM curves for cumulative MACE up to 10-year follow-up stratified by *P*_d_/*P*_a_ and FFR agreement. A normal *P*_d_/*P*_a_ was generally associated with a favourable clinical outcome, regardless of the accompanying FFR value, whereas abnormal *P*_d_/*P*_a_ was generally associated with an impaired clinical outcome, regardless of the accompanying FFR value (overall Breslow *p* = 0.04).

### Agreement with CFR

CFR was significantly different across the four quadrants of *P*_d_/*P*_a_ and FFR (dis)agreement (Tab. 3). A normal *P*_d_/*P*_a_ was generally associated with high CFR and an abnormal *P*_d_/*P*_a_ was generally associated with CFR values around the ischaemic cut-off, regardless of the accompanying FFR value (overall *p* = 0.006).

**Table 3:** Study population characteristics according to FFR—*P*_d_/*P*_a_ disagreement

		FFR > 0.80 and *P*_d_*P*_d_/*P*_a_ > 0.92	FFR ≤ 0.80 and *P*_d_/*P*_a_ > 0.92		FFR > 0.80 and *P*_d_/*P*_a_ ≤ 0.92	FFR ≤ 0.80 and *P*_d_/*P*_a_ ≤ 0.92	Overall *p*-value
Number of patients		72	29		15	38	
*Demographics*							
	Age, years	61 ± 11	57 ± 11		66 ± 8	60 ± 11	0.09
	Male gender, *n* (%)	51 (71)	22 (76)		7 (47)	30 (78)	0.14
*Coronary risk factors*							
	Hypertension, *n* (%)	43 (60)	8 (28)		9 (60)	14 (37)	0.22
	Hyperlipidaemia, *n* (%)	41 (57)	17 (59)		8 (53)	23 (61)	0.97
	Positive family history, *n* (%)	33 (46)	14 (48)		9 (60)	20 (53)	0.76
	Cigarette smoking, *n* (%)	20 (28)	9 (31)		2 (13)	17 (45)	0.13
	Diabetes mellitus, *n* (%)	9 (13)	8 (28)		3 (20)	4 (11)	0.2
	Prior myocardial infarction, *n* (%)	33 (46)	8 (28)		3 (20)	13 (34)	0.15
	Prior PCI, *n* (%)	20 (28)	8 (28)		2 (13)	4 (11)	0.14
*Medication at hospital admission*							
	Beta-blocker, *n* (%)	57 (79)	24 (83)		10 (67)	29 (76)	0.63
	Nitrates, *n* (%)	51 (71)	19 (66)		12 (80)	28 (74)	0.8
	Calcium antagonists, *n* (%)	44 (61)	17 (59)		11 (73)	29 (76)	0.31
	ACE inhibitors, *n* (%)	15 (21)	2 (7)		2 (13)	9 (24)	0.28
	Lipid-lowering drugs, *n* (%)	39 (54)	15 (52)		9 (60)	24 (63)	0.77
	Acetylsalicylic acid, *n* (%)	69 (96)	29 (100)		15 (100)	36 (95)	0.76
*Angiographic characteristics*							
	Diameter stenosis, %	53 (44–57)		55 (50–62)	51 (47–56)	55 (50–58)	0.46
	Reference diameter, mm	3.0 (2.6–3.5)		2.7 (2.5–3.3)	2.8 (2.5–3.0)	2.6 (2.3–3.1)	0.09
	Minimal lumen diameter, mm	1.5 (1.1–1.7)		1.2 (1.1–1.6)	1.3 (1.1–1.5)	1.2 (1.1–1.3)	0.03
*Physiological characteristics*							
	APV basal, cm/s	15 (12–18)		18 (13–24)	16 (13–24)	17 (11–21)	0.19
	APV hyperaemia, cm/s	38 (31–48)		38 (31–55)	38 (29–54)	36 (30–45)	0.68
	CFR	2.6 (2.2–3.0)		2.5 (1.9–2.9)	2.2 (1.8–2.8)	2.2 (1.8–2.7)	0.006
	FFR	0.89 (0.85–0.93)		0.78 (0.73–0.78)	0.85 (0.82–0.87)	0.71 (0.67–0.76)	0.001
	*P*_d_/*P*_a_	0.97 (0.96–0.98)		0.94 (0.94–0.96)	0.91 (0.91–0.92)	0.89 (0.85–0.91)	0.001

Values are number (%), mean ± standard deviaion, median (1st, 3rd quartile)

*FFR* fractional flow reserve; *P*_*d*_*/P*_*a*_ resting distal coronary-to-aortic pressure ratio; *PCI* percutaneous coronary intervention; *ACE* angiotensin converting enzyme; *APV* average peak flow velocity; *CFR* coronary flow reserve

## Discussion

The current article is the first to document that *P*_d_/*P*_a_ demonstrates a continuous and independent relationship with subsequent long-term clinical outcomes which is at least equivalent to that of FFR, and that *P*_d_/*P*_a_ exceeds FFR as a risk stratification tool at the contemporary clinical cut-off values. When discordance with FFR occurs, *P*_d_/*P*_a_ may therefore confer superior clinical value.

### Prognostic relevance of resting versus hyperaemic stenosis pressure drops

The superior prognostic relevance of resting *P*_d_/*P*_a_ over FFR at contemporary cut-offs may be explained by a better agreement of *P*_d_/*P*_a_ with coronary flow. Our observations support those of previous studies that demonstrated a better relationship between resting indices and Doppler-derived CFR than for FFR and Doppler-derived CFR [3, 6, 7]. It has been documented that, when FFR disagrees with CFR, CFR has superior prognostic value over FFR for long-term clinical outcomes [8, 9, 15]. This is likely due to the fact that FFR and CFR move in opposite directions from resting conditions to hyperaemia: FFR decreases and becomes more abnormal, while CFR increases and becomes more normal. Hence, the combination of an abnormal FFR and normal *P*_d_/*P*_a_ may occur on the basis of a normal *P*_d_/*P*_a_ value that decreases to abnormal FFR values at hyperaemia due to a large increase in coronary flow with a normal CFR. Such stenoses are considered non-flow-limiting, and likely have excellent clinical outcomes when managed medically [8, 9, 15, 17, 18]. On the other hand, an abnormal *P*_d_/*P*_a_ may occur in combination with a normal FFR when a stenosis may coexist with an exhausted CFR as a result of compensatory vasodilation during resting conditions to maintain resting perfusion. The abnormal *P*_d_/*P*_a_ may only decrease to normal FFR values due to the limited increase in coronary flow from baseline to hyperaemia. The optimal management of vessels exhibiting this haemodynamic pattern remains a matter of debate [19]. Since myocardial function thrives on coronary flow and not on perfusion pressure, reductions in distal coronary perfusion, however, should not be associated with impaired myocardial function as long as adequate coronary flow is present [20]. This is supported by a recent randomised study documenting excellent clinical outcomes when magnetic resonance imaging-defined perfusion deficits were used to guide coronary intervention [18]. Since myocardial function and clinical outcomes are determined by coronary flow independent of coronary pressure [8, 9, 15, 20], and *P*_d_/*P*_a_ has better agreement with invasively measured coronary flow than FFR [3], this may provide an explanation why *P*_d_/*P*_a_ has superior prognostic value when there is disagreement with FFR.

### Comparison with previous FFR studies

Importantly, our observation that there was no difference in clinical outcomes between FFR-positive and FFR-negative cases might wrongly be interpreted to be in contrast with the findings from FAME (Fractional Flow Reserve Versus Angiography for Multivessel Evaluation) and FAME II [16, 21]. FAME compared FFR versus angiography for guidance of revascularisation and documented superior clinical outcome using a FFR-guided revascularisation strategy. FAME II compared PCI + optimal medical therapy (OMT) versus OMT for treatment of FFR-positive lesions and documented superior clinical outcome for lesions treated with PCI + OMT. It is important to note that the average FFR in FFR-positive stenosis in FAME was 0.60 ± 0.14 [21]. In FAME II, the average FFR in FFR-positive stenoses amounted to 0.68 ± 0.10 in the medical therapy group and 0.68 ± 0.15 in the PCI group [16]. Hence, the stenoses included in the FAME studies were physiologically much more severe than those encountered typically in contemporary daily practice. Most importantly, among these severe stenoses in the FAME II study, around 60% of lesions in the medical therapy group did not require any coronary intervention within 2 years after initial deferral from revascularisation—a result that is consistent up to 5‑year follow-up. The present study involves true physiologically intermediate stenoses as typically encountered in daily practice (Tab. 1; [2, 10, 11, 18]). This is relevant for the observed differences in clinical outcomes compared with the FAME studies: FFR reflects a risk continuum, where risk for adverse events becomes higher with decreasing FFR [22]. This risk continuum for FFR was also noted in the Cox regression analysis for FFR in the present study. However, the FAME II trial showed that the benefit of revascularisation was supreme in vessels with FFR **<** 0.65. Similarly, a recent patient-level meta-analysis on the prognostic value of FFR identified an optimal 0.67 FFR threshold for clinical outcomes [22]. These findings support the lack of a difference in MACE between FFR ≤ 0.80 and > 0.80 in the present study, where, similar to daily clinical practice, most FFR values in FFR-positive stenoses were >0.70 and therefore above the FFR thresholds associated with impaired clinical outcomes.

### Limitations

The relatively small sample size limits the statistical power and the strength of the conclusions, especially for the discordant groups. We used intracoronary administration of 20–40 µg of adenosine, which remains a subject for debate. However, it has been documented that the observed FFR is already within 0.02 of its minimal value at intracoronary adenosine dosages above 23 µg [23]. Since the test/retest repeatability of FFR itself has a standard deviation of 0.02 [24], FFR differences **<** 0.02 are smaller than the variability of the FFR measurement itself and, therefore, are considered clinically irrelevant [23]. It is therefore unlikely that the dose of adenosine has interfered with the results in the present study. This is supported by the limited differences in FFR values derived from low-dose intracoronary versus intravenous adenosine administration [25], and by the fact that such small differences in FFR between intravenous and intracoronary adenosine were documented to be clinically irrelevant in the DEFER trial [26]. Additionally, this study is limited by the assessment of adverse events at long-term follow-up partly performed by telephone contact. Such an approach is sensitive towards patient recall bias, which may result in under-reporting of adverse events. Nonetheless, the long-term MACE rates reported in the present study are comparable with those reported in contemporary studies using FFR guidance [26, 27].

## Conclusion

The present study documents that *P*_d_/*P*_a_ demonstrates a continuous and independent relationship with subsequent long-term clinical outcomes which is at least equivalent to that of FFR, and suggests that *P*_d_/*P*_a_ exceeds FFR as a risk stratification tool at its contemporary cut-off value.

## Caption Electronic Supplementary Material

Instantaneous wave-free ratio (iFR) sub-analysis: long-term prognostic implications of iFR compared with FFR

## References

[1] Sen S, Escaned J, Malik IS (2012). Development and validation of a new adenosine-independent index of stenosis severity from coronary wave-intensity analysis: results of the ADVISE (ADenosine Vasodilator Independent Stenosis Evaluation) study. J Am Coll Cardiol.

[2] Jeremias A, Maehara A, Généreux P (2014). Multicenter core laboratory comparison of the instantaneous wave-free ratio and resting Pd/Pa with fractional flow reserve: the RESOLVE study. J Am Coll Cardiol.

[3] Echavarría-Pinto M, van de Hoef TP, van Lavieren MA (2015). Combining baseline distal-to-aortic pressure ratio and fractional flow reserve in the assessment of coronary stenosis severity. JACC Cardiovasc Interv.

[4] Hwang D, Jeon K-H, Lee JM (2017). Diagnostic performance of resting and hyperemic invasive physiological indices to define myocardial ischemia: validation with (13)N-ammonia positron emission tomography. JACC Cardiovasc Interv.

[5] de Waard GA, Danad I, Petraco R (2018). Fractional flow reserve, instantaneous wave-free ratio, and resting Pd/Pa compared with [15O]H2O positron emission tomography myocardial perfusion imaging: a PACIFIC trial sub-study. Eur Heart J.

[6] Petraco R, van de Hoef TP, Nijjer S (2014). Baseline instantaneous wave-free ratio as a pressure-only estimation of underlying coronary flow reserve: results of the JUSTIFY-CFR Study (Joined Coronary Pressure and Flow Analysis to Determine Diagnostic Characteristics of Basal and Hyperemic Indices of Functional Lesion Severity-Coronary Flow Reserve). Circ Cardiovasc Interv.

[7] Cook CM, Jeremias A, Petraco R (2017). Fractional flow reserve/instantaneous wave-free ratio discordance in angiographically intermediate coronary stenoses: an analysis using Doppler-derived coronary flow measurements. JACC Cardiovasc Interv.

[8] van de Hoef TP, van Lavieren MA, Damman P (2014). Physiological basis and long-term clinical outcome of discordance between fractional flow reserve and coronary flow velocity reserve in coronary stenoses of intermediate severity. Circ Cardiovasc Interv.

[9] Lee JM, Jung J-H, Hwang D (2016). Coronary flow reserve and microcirculatory resistance in patients with intermediate coronary stenosis. J Am Coll Cardiol.

[10] Davies JE, Sen S, Dehbi H-M (2017). Use of the instantaneous wave-free ratio or fractional flow reserve in PCI. N Engl J Med.

[11] Götberg M, Christiansen EH, Gudmundsdottir IJ (2017). Instantaneous wave-free ratio versus fractional flow reserve to guide PCI. N Engl J Med.

[12] Meuwissen M, Siebes M, Chamuleau SAJ (2002). Hyperemic stenosis resistance index for evaluation of functional coronary lesion severity. Circulation.

[13] Meuwissen M, Chamuleau SAJ, Siebes M (2008). The prognostic value of combined intracoronary pressure and blood flow velocity measurements after deferral of percutaneous coronary intervention. Catheter Cardiovasc Interv.

[14] Chamuleau SAJ, Tio RA, De Cock CC (2002). Prognostic value of coronary blood flow velocity and myocardial perfusion in intermediate coronary narrowings and multivessel disease. J Am Coll Cardiol.

[15] van de Hoef TP, Echavarría-Pinto M, van Lavieren MA (2015). Diagnostic and prognostic implications of coronary flow capacity: a comprehensive cross-modality physiological concept in ischemic heart disease. JACC Cardiovasc Interv.

[16] De Bruyne B, Fearon WF, Pijls NHJ (2014). Fractional flow reserve-guided PCI for stable coronary artery disease. N Engl J Med.

[17] Murthy VL, Naya M, Foster CR (2011). Improved cardiac risk assessment with noninvasive measures of coronary flow reserve. Circulation.

[18] Nagel E, Greenwood JP, McCann GP (2019). Magnetic resonance perfusion or fractional flow reserve in coronary disease. N Engl J Med.

[19] van Lavieren MA, van de Hoef TP, Sjauw KD (2015). How should I treat a patient with refractory angina and a single stenosis with normal FFR but abnormal CFR?. EuroIntervention.

[20] Smalling RW, Kelley K, Kirkeeide RL, Fisher DJ (1985). Regional myocardial function is not affected by severe coronary depressurization provided coronary blood flow is maintained. J Am Coll Cardiol.

[21] Tonino PAL, De Bruyne B, Pijls NHJ (2009). Fractional flow reserve versus angiography for guideing percutaneous coronary intervention. N Engl J Med.

[22] Johnson NP, Tóth GG, Lai D (2014). Prognostic value of fractional flow reserve: linking physiologic severity to clinical outcomes. J Am Coll Cardiol.

[23] Adjedj J, Toth GG, Johnson NP (2015). Intracoronary adenosine: dose-response relationship with hyperemia. JACC Cardiovasc Interv.

[24] Johnson NP, Jeremias A, Zimmermann FM (2016). Continuum of vasodilator stress from rest to contrast medium to adenosine hyperemia for fractional flow reserve assessment. JACC Cardiovasc Interv.

[25] Rigattieri S, Biondi Zoccai G, Sciahbasi A (2017). Meta-analysis of head-to-head comparison of intracoronary versus intravenous adenosine for the assessment of fractional flow reserve. Am J Cardiol.

[26] Bech GJ, De Bruyne B, Pijls NH (2001). Fractional flow reserve to determine the appropriateness of angioplasty in moderate coronary stenosis: a randomized trial. Circulation.

[27] Li J, Elrashidi MY, Flammer AJ (2013). Long-term outcomes of fractional flow reserve-guided vs. angiography-guided percutaneous coronary intervention in contemporary practice. Eur Heart J.

