# Supplementary material for: Prognostic implications of resting distal coronary-to-aortic pressure ratio compared with fractional flow reserve: a 10-year follow-up study after deferral of revascularisation
Source: Neth Heart J. 2020 Jan 21;28(2):96–103. doi: 10.1007/s12471-020-01365-6 (PMC6977812; doi:10.1007/s12471-020-01365-6)

**Supplementary data: Instantaneous wave-free ratio (iFR) sub-analysis.**

Instantaneous wave-free-ratio (iFR) was calculated only in cases where raw pulsatile coronary pressure tracings obtained >30 seconds after last contrast-medium injection were available, using a custom software package written by Imperial College London, UK, in Matlab® (Mathworks Inc, Matick MA). IFR was defines as the distal to aortic pressure ratio measured within the wave-free period, and iFR≤0.89 was considered abnormal.

iFR could be calculated in 44 patients with 51 stenoses: MACE analyses included 44 patients and 44 stenoses. Median iFR was 0.91 (Q1, Q2: 0.87, 0.94) and 39.0% of stenoses were hemodynamically significant according to iFR. In the sub-population where iFR was available, iFR and P_d_/P_a_ were significantly and equivalently associated with long-term MACE, whereas there was no association between FFR and long-term MACE (supplementary table 1)(iFR sHR: 0.66 (95% CI: 0.46–0.95), *p*=0.026; P_d_/P_a_ sHR: 0.69 (95% CI: 0.48-1.00), *p*=0.052; FFR sHR 0.80 (95% CI: 0.49-1.32), p=0.39). Stenoses with an abnormal iFR had a significantly higher KM-estimate of MACE compared with stenoses with a normal iFR (iFR≤0.89: 52.9%, vs iFR>0.89: 22.2%; Breslow *p*=0.007)(supplementary figure 1), IFR and FFR agreed in 66% of cases (29 out of 44): 23% of stenoses (10 out of 44) were concordant abnormal, and 43% of stenoses (19 out of 44) were concordant normal. Discordance occurred in 34% of cases (15 out of 44), and was characterized by normal FFR and abnormal iFR in 16% of cases (7 out of 44), and by abnormal FFR and normal iFR in 18% of cases (8 out of 44) (supplementary figure 2). Supplementary figure 3 shows the KM curves for cumulative MACE up to 10-year follow-up. A normal iFR was generally associated with a favorable clinical outcome, regardless of the accompanying FFR value, whereas an abnormal iFR was generally associated with an impaired clinical outcome regardless of the accompanying FFR value (overall Breslow *p*=0.004)

| **Supplementary table 1: Univariate and adjusted Cox-regression analyses for long-term MACE** | | | | | |  |  |  |  |  |  |  |  |  |  |  |  |  |
| --- | --- | --- | --- | --- | --- | --- | --- | --- | --- | --- | --- | --- | --- | --- | --- | --- | --- | --- |
| iFR-sub-population (n=44) | | | | | |  |  |  |  |  |  |  |  |  |  |  |  |  |
| Univariate analysis | | | Adjusted analysis* | | |  |  |  |  |  |  |  |  |  |  |  |  |  |
| Variable | sHR (95%CI) | *P* value | Variable | sHR (95%CI) | *P* value |  |  |  |  |  |  |  |  |  |  |  |  |  |
| P_d_/P_a_ | 0.69(0.50-0.94) | 0.020 | P_d_/P_a_ | 0.69(0.48-1.00) | 0.052 |  |  |  |  |  |  |  |  |  |  |  |  |  |
| FFR | 0.78(0.48-1.27) | 0.314 | FFR | 0.80(0.49-1.32) | 0.385 |  |  |  |  |  |  |  |  |  |  |  |  |  |
| iFR | 0.82(0.47-0.89) | 0.007 | iFR | 0.66(0.46-0.95) | 0.026 |  |  |  |  |  |  |  |  |  |  |  |  |  |
| *adjusted for angiotensin converting enzyme inhibitor use, the presence of diabetes mellitus, and age at the time of physiological assessment. | | | | | |  | | | |  |  |  |  |  |  |  |  |  |
| Data presented as standardized hazard ratio and its 95% confidence interval | | | | | |  |  |  |  |  |  |  |  |  |  |  |  |  |
| sHR = standardized hazard ratio; P_d_/P_a_ = resting distal coronary to aortic pressure ratio; FFR = fractional flow reserve; iFR = instantaneous wave-free ratio | | | | | |  | |  |  |  |  |  |  |  |  |  |  |  |

| **Supplementary table 2. Study population characteristics according to FFR - iFR discordance** | | | | | |  | |  | |  | | |
| --- | --- | --- | --- | --- | --- | --- | --- | --- | --- | --- | --- | --- |
|  | |  | **FFR>0**.**80 & iFR>0**.**89** | **FFR≤0**.**80 & iFR>0**.**89** | **FFR>0**.**80 & iFR≤0**.**89** | | **FFR≤0**.**80 & iFR≤0**.**89** | | **Overall p-value** | |  |  |
| Number of patients | | | 19 | 8 | 7 | | 10 | |  | |  |  |
| *Demographics* | | |  |  |  | |  | |  | |  |  |
|  | | Age, yrs | 59 ± 11 | 60 ± 10 | 68 ± 9 | | 55 ± 15 | | 0.16 | |  |  |
|  | | Male, n(%) | 13 (68) | 6 (75) | 2 (30) | | 8 (80) | | 0.17 | |  |  |
| *Coronary risk factors* | | |  |  |  | |  | |  | |  |  |
|  | | Hypertension, n(%) | 9 (47) | 2 (25) | 4 (57) | | 3 (30) | | 0.52 | |  |  |
|  | | Hyperlipidemia, n(%) | 9 (47) | 6 (75) | 5 (71) | | 5 (50) | | 0.51 | |  |  |
|  | | Positive family history, n(%) | 13 (68) | 4 (50) | 5 (71) | | 5 (50) | | 0.63 | |  |  |
|  | | Cigarette smoking, n(%) | 5 (26) | 2 (25) | 1 (14) | | 6 (60) | | 0.22 | |  |  |
|  | | Diabetes mellitus, n(%) | 2 (11) | 1 (13) | 2 (29) | | 2 (20) | | 0.71 | |  |  |
|  | | Prior myocardial infarction, n(%) | 7 (37) | 2 (25) | 2 (29) | | 2 (20) | | 0.85 | |  |  |
|  | | Prior PCI, n(%) | 1 (5) | 1 (13) | 1 (14) | | 3 (30) | | 0.29 | |  |  |
| *Medication at hospital admission* | | |  |  |  | |  | |  | |  |  |
|  | | Beta-blocker, n(%) | 17 (89) | 6 (75) | 7 (100) | | 8 (80) | | 0.53 | |  |  |
|  | | Nitrates, n(%) | 15 (79) | 6 (75) | 7 (100) | | 7 (70) | | 0.56 | |  |  |
|  | | Calcium antagonists, n(%) | 12 (63) | 6 (75) | 4 (57) | | 7 (70) | | 0.89 | |  |  |
|  | | ACE-inhibitors, n(%) | 5 (26) | 1 (13) | 1 (14) | | 2 (20) | | 0.95 | |  |  |
|  | | Lipid-lowering drugs, n(%) | 10 (53) | 4 (50) | 4 (57) | | 7 (70) | | 0.82 | |  |  |
|  | | Aspirin, n(%) | 18 (95) | 8 (100) | 7 (100) | | 10 (100) | | 1 | |  |  |
| *Angiographic characteristics* | | |  |  |  | |  | |  | |  |  |
|  | | Diameter stenosis, % | 52 (44 - 57) | 54 (50 - 58) | 43 (42 - 53) | | 53 (51 - 68) | | 0.28 | |  |  |
|  | | Reference diameter, mm | 2.8 (2.7 - 3.2) | 3.2 (2.7 - 3.3) | 2.6 (2.1 - 3.1) | | 2.6 (1.9 - 2.7) | | 0.06 | |  |  |
|  | | Minimal lumen diameter, mm | 1.5 (1.2 - 1.7) | 1.3 (1.2 - 1.6) | 1.4 (1.0 - 1.6) | | 1.0 (0.9 - 1.2) | | 0.09 | |  |  |
| *Physiological characteristics* | | |  |  |  | |  | |  | |  |  |
|  | APV resting, cm/s | | 15 (12 - 18) | 18 (13 - 24) | 16 (13 - 24) | | 17 (11 - 21) | | 0.19 | |  |  |
|  | APV hyperemia, cm/s | | 38 (31 - 48) | 38 (31 - 55) | 38 (29 - 54) | | 36 (30 - 45) | | 0.68 | |  |  |
|  | CFR | | 2.6 (2.2 - 3.0) | 2.5 (1.9 - 2.9) | 2.2 (1.8 - 2.8) | | 2.2 (1.8 - 2.7) | | 0.006 | |  |  |
|  | FFR | | 0.87 (0.84 - 0.92) | 0.76 (0.72 - 0.80) | 0.84 (0.81 - 0.87) | | 0.69 (0.65 - 0.76) | | - | |  |  |
|  | P_d_/P_a_ | | 0.96 (0.94 - 0.98) | 0.94 (0.93 - 0.95) | 0.93 (0.91 - 0.97) | | 0.88 (0.81 - 0.92) | | <0.001 | |  |  |
|  | iFR | | 0.94 (0.91 - 0.98) | 0.92 (0.92 - 0.95) | 0.88 (0.82 - 0.89) | | 0.85 (0.74 - 0.85) | | - | |  |  |
| Values are mean ± SD, median (Q1 - Q3) or n (%) | | | |  | |  | |  | |  | | |
| FFR = fractional flow reserve; iFR = instantaneous wave-free ratio; PCI = percutaneous coronary intervention; P_d_/P_a_ = resting; distal coronary to aortic pressure ratio | | | | | | | | | | | |  |

Supplementary figure 1


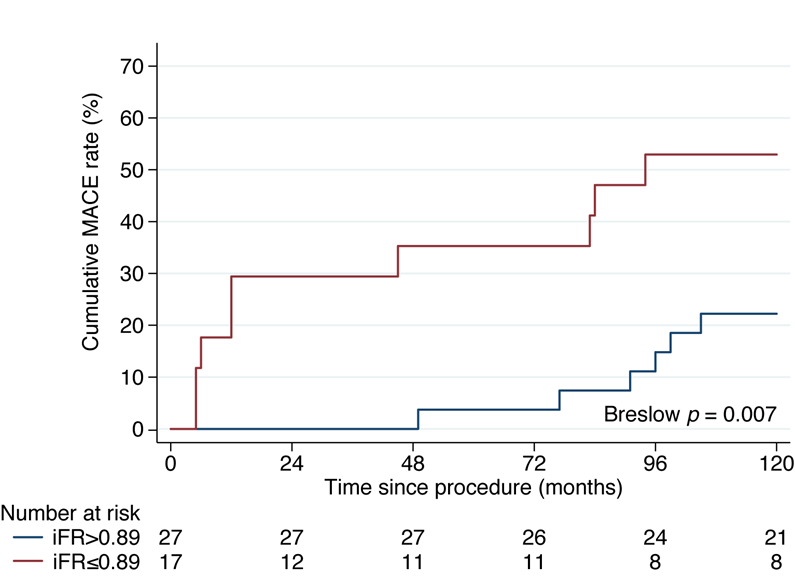


Supplementary figure 2


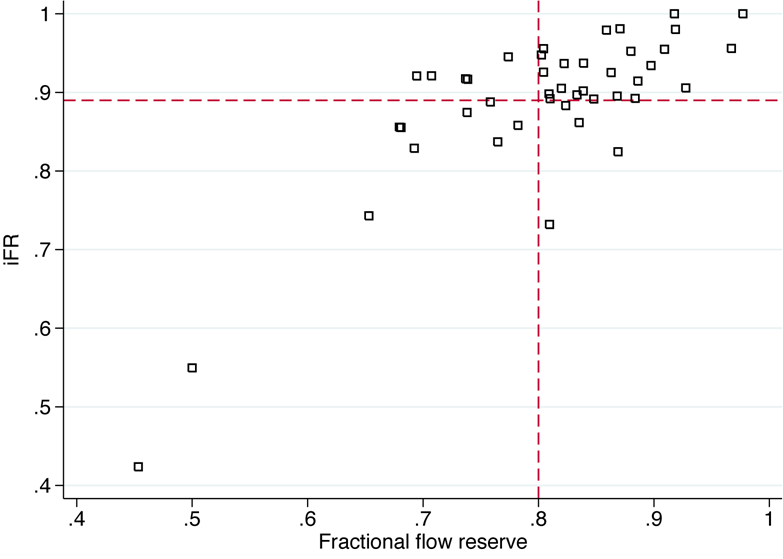


Supplementary figure 3


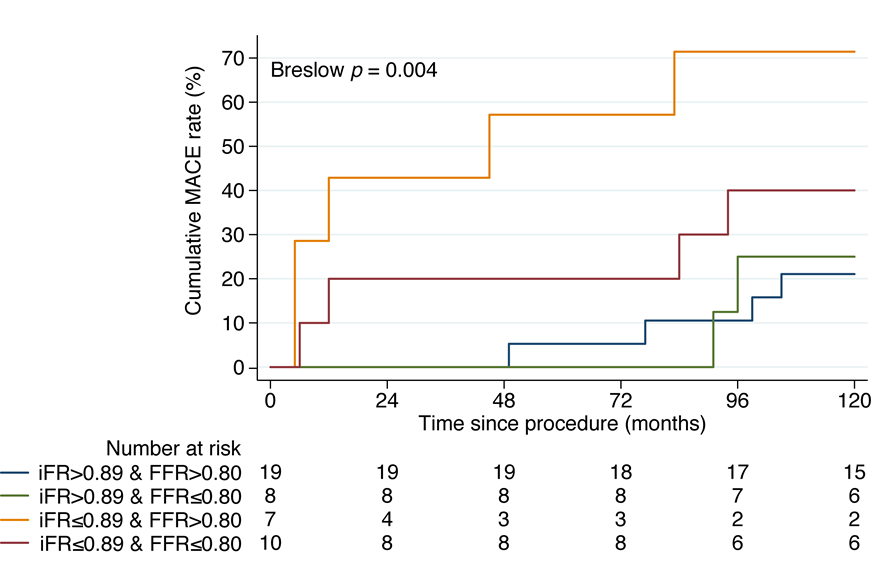

Supplement: Supplementary file 1 — Instantaneous wave-free ratio (iFR) sub-analysis: long-term prognostic implications of iFR compared with FFR [file 12471_2020_1365_MOESM1_ESM.docx]
